# What Enables and Constrains the Inclusion of the Social Determinants of Health Inequities in Government Policy Agendas? A Narrative Review

**DOI:** 10.15171/ijhpm.2017.130

**Published:** 2017-11-11

**Authors:** Phillip Baker, Sharon Friel, Adrian Kay, Fran Baum, Lyndall Strazdins, Tamara Mackean

**Affiliations:** ^1^Institute for Physical Activity and Nutrition, School of Exercise and Nutrition Sciences, Deakin University, Geelong, VIC, Australia.; ^2^School of Regulation and Global Governance (RegNet), College of Asia and the Pacific, Australian National University, Canberra, Australia.; ^3^Institute of Policy Studies, University Brunei Darussalam, Gadong, Brunei Darussalam.; ^4^Southgate Institute of Health, Society and Equity, Flinders University, Adelaide, SA, Australia.; ^5^National Centre for Epidemiology and Population Health, College of Medicine, Biology & Environment, Australian National University, Canberra, Australia.

**Keywords:** Health Inequities, Health Inequalities, Social Determinants of Health, Agenda-Setting, Policy Process

## Abstract

**Background:** Despite decades of evidence gathering and calls for action, few countries have systematically attenuated health inequities (HI) through action on the social determinants of health (SDH). This is at least partly because doing so presents a significant political and policy challenge. This paper explores this challenge through a review of the empirical literature, asking: what factors have enabled and constrained the inclusion of the social determinants of health inequities (SDHI) in government policy agendas?

**Methods:** A narrative review method was adopted involving three steps: first, drawing upon political science theories on agenda-setting, an integrated theoretical framework was developed to guide the review; second, a systematic search of scholarly databases for relevant literature; and third, qualitative analysis of the data and thematic synthesis of the results. Studies were included if they were empirical, met specified quality criteria, and identified factors that enabled or constrained the inclusion of the SDHI in government policy agendas.

**Results:** A total of 48 studies were included in the final synthesis, with studies spanning a number of country-contexts and jurisdictional settings, and employing a diversity of theoretical frameworks. Influential factors included the ways in which the SDHI were framed in public, media and political discourse; emerging data and evidence describing health inequalities; limited supporting evidence and misalignment of proposed solutions with existing policy and institutional arrangements; institutionalised norms and ideologies (ie, belief systems) that are antithetical to a SDH approach including neoliberalism, the medicalisation of health and racism; civil society mobilization; leadership; and changes in government.

**Conclusion:** A complex set of interrelated, context-dependent and dynamic factors influence the inclusion or neglect of the SDHI in government policy agendas. It is better to think about these factors as increasing (or decreasing) the ‘probability’ of health equity reaching a government agenda, rather than in terms of ‘necessity’ or ‘sufficiency.’ Understanding these factors may help advocates develop strategies for generating political priority for attenuating HI in the future.

## Background

In recent decades there have been numerous national and international calls to reduce health inequities (HI) through action on the social determinants of health (SDH): the conditions in which people are born, grow, live, work and age, which are in turn shaped by the distribution of power, money and resources within and between countries.^1,2^ In 2008, the report of the World Health Organization’s (WHO’s) Commission on the Social Determinants of Health acknowledged the critical role of political action;

This is a long-term agenda, requiring … major changes in social policies, economic arrangements, and political action … The knowledge and the means to change are at hand … What is needed now is the political will to implement these eminently difficult but feasible changes.^1^

Yet despite decades of evidence gathering and calls for action, in very few countries has the social determinants of health inequities (SDHI) emerged onto government policy agendas. To the contrary, despite gains in overall life expectancy existing policy approaches appear to be widening, rather than attenuating, HI in many countries.^3^ This is at least partly because reducing HI – the avoidable or remediable differences in the health of population groups, whether defined economically, socially, demographically or geographically – presents a significant political challenge.^4^

This paper explores this political challenge through a review of the empirically-based literature. The aim is to identify factors that have enabled and constrained the inclusion of health equity in government policy agendas. Although there are previous reviews of this nature these have tended to focus on only a small number of studies,^5^ on advocacy processes,^6^ or on theoretical rather than on empirical literature.^4^

In this paper we make use of insights from the political and policy sciences, which argue that policy-making is a complex, non-linear process, where problems are not always easily-definable. Evidence can and does inform government policy agendas, but it can also be lacking, contested or politicised.^8^ Decision-makers are seen as having ‘bounded rationality’ due to pre-existing cognitive biases as well as limited time, processing capacity and resources to analyse problems and formulate solutions systematically.^9^ Furthermore, while government priorities can be shaped by evidence, they are also shaped by the values and interests of powerful interest groups, the ideas they use to interpret and portray issues, and the extent to which such portrayals resonate with existing belief systems (ie, ideologies), institutional structures and historical policy trajectories.^4,10,11^

Tackling HI is an innately political undertaking because the conditions of everyday life that are important for health are strongly influenced by the distributional policies of governments – those that determine the allocation of resources, money and power between countries and the political constituencies within them.^12^ As Harold Laswell wrote, distributional decisions over “who gets what, when and how” are at the heart of political decision-making.^13^ The concepts of equity, fairness and social justice are also highly contested in political debates over distributional decisions, reflecting the ideological lenses through which different interest groups come to understand and portray public policy issues (ie, these are explicitly normative concepts, as distinct from implicitly normative descriptors like health inequalities, disparities and gradients used in social epidemiology).^14^

Additionally, the nature of the SDHI policy problem is by definition a ‘wicked’ one. As Exworthy describes it,^4^ the complex causal pathways linking the SDH and HI are mismatched with political preferences for simple policy problems that are solvable using existing policy instruments and institutional arrangements. Needed policy actions often fall outside of health portfolios requiring coordinated actions across many sectors, at multiple levels, within and outside of government. This can bring competing interests and worldviews into play and confers a certain lack of ‘institutional ownership.’ Furthermore, the ‘slow-burning’ determinants of HI, particularly those that accumulate over the life-course or those of an inter-generational nature, are often out of sync with ‘fast-burn’ policy crises, election cycles and legislative agendas geared towards quick wins.^4^

A political science approach can inform an understanding as to how such challenges might be overcome, why the SDHI has so far ‘failed to launch’ onto government policy agendas, and how political priority might come about in future. To do this we draw upon theories of the policy process. This process is often described as comprising a series of stages including problem identification, agenda setting, policy formation, adoption, implementation, and evaluation.^15^ Although this is an overly-simplistic representation of how policy is actually made,^4^ the early stages are critical to generating political priority (but not necessarily for sustaining it). The focus of this paper is on the problem identification and agenda-setting phases when issues come to be defined and recognised as policy problems, whereas others are neglected, ignored, or deliberately ‘organized out’ of politics.^10,15,16^

## Methods

A narrative review method was adopted because of the complex, multi-factorial nature of the topic (making statistical meta-analysis inappropriate), as well as the qualitative methods typically used in the SDHI policy literature (making a narrative textual account of the findings suitable).^17,18^ This involved three steps: first, the development of an integrated theoretical framework to guide the search and analysis; second, a systematic search for relevant literature and quality appraisal; and third, analysis of the data and thematic synthesis of the results.

This review was undertaken by a team with expertise in political science and public health. It informs a broader project on agenda-setting and the SDHI as part of the Centre for Research Excellence on the Social Determinants of Heath Equity (CRESDHE), funded by the Australian National Health and Medical Research Council.^19^ An important focus of this work is addressing the 11-year gap in life expectancy between Aboriginal and Torres Strait Islander peoples and other Australians; we therefore included Indigenous health related terms in our search. This decision was also driven by the existence of very large life expectancy gaps between Indigenous peoples and others globally.^20^

### Integrated Theoretical Framework

Acknowledging that theoretical pluralism (ie, using multiple theories rather than one alone) can help to inform a more robust understanding of political and policy phenomena,^21^ two theoretical frameworks were integrated to guide the search and analysis. These were chosen for their relevance (ie, explicit focus on agenda-setting, political priority, and policy change), frequency of use in the public health policy literature, and complementariness (ie, together they offer a coherent and more complete set of theorised factors).

First is Kingdon’s multiple-streams framework. This assumes that policy-makers operate under conditions of ambiguity and bounded rationality, and recognises the importance of timing in the policy process. Kingdon defines an agenda as “the list of subjects or problems which governmental officials, and people outside of government closely associated with those officials, are paying some serious attention at any given time” (p. 3).^10^ Kingdon maintains that for an issue to receive priority on a government agenda three independent streams must converge: the problem stream, where an issue is perceived as a problem worthy of addressing; the policy stream, where a number of alternative policy solutions are proposed to address the problem; and the politics stream, where political events (eg, the election of a new government, swings in public opinion, and interest group mobilization), create opportunities for policy reform. The problem stream is concerned with the problem identification stage of the policy process where issues come to be defined, portrayed and politicized in public discourse.^10^ During certain time periods known as ‘policy windows’ these three streams can converge and the probability of an issue ‘launching’ onto a government agenda greatly increases. This convergence can be facilitated by ‘policy entrepreneurs,’ usually highly-capable and politically-savvy technical experts, and supported by ‘policy advocates,’ typically high-profile actors such as heads of government or other high-level officials. However, not all issues that governments come to constitute as ‘problems’ will reach what Kingdon calls the ‘decision agenda,’ the smaller list of issues considered for policy and legislative enactment.^10^ Thus, it is important to consider how problemitisations change as issues travel through the agenda-setting process and on into policy.

Shiffman and Smith build upon Kingdon’s framework to focus specifically on priority-setting in global and national health.^11,16^ In this perspective, issues do not simply exist ‘out there’ as objective entities waiting to be addressed; rather they come to be constructed as problems through interactions among political actors. Thus, there can be many competing interpretations regarding an issues definition, what causes it, who or what is responsible for it, and what the solutions should be. Their framework proposes a number of variables that increase the probability of health issues receiving priority in government agendas. Policy communities, defined as networks of individuals and organizations who share a common concern for an issue, play an important role. These communities are more likely to advance their issue when they are cohesive, led by champions for the cause, and when they frame (ie, publically portray) the problem and solutions in ways that resonate with the beliefs and ideologies of political decision-makers. Importantly, the framework acknowledges the importance of governance structures, including the role of norms (dominant belief systems and practices) and the institutions that enforce these norms within a given sector.

These theories focus primarily on the early stages of the policy process. However, they also acknowledge the blurred boundaries between stages, particularly with regard to how issues travel onto and through the agenda-setting stage and into policy. For example, the selection of policy alternatives during agenda-setting can have important policy design implications (ie, agenda-setting policy-design interactions). Some issues, once on the agenda, may be maintained there and periodically re-examined.

### Search Strategy and Process

A search for primary literature was conducted between July and August 2016. A detailed search diary was kept to record progress and any modifications to the search strategy (Text S1). Four scholarly databases were searched: PubMed, Scopus, ProQuest, and ISI Web of Science. These databases were selected for their relevance and comprehensiveness after consultation with two Australian National University librarians trained in systematic search. The search terms given in Table 1 were generated from three sources. Problem identification and agenda-setting terms were identified from the integrated theoretical framework outlined earlier and associated literature.^10,11,16^ General SDH and HI terms were identified from the WHO’s Commission on the Social Determinants of Health report and further clarified from glossaries on social epidemiology and health inequalities.^22-24^ Terms for specific social determinants (eg, education, gender, and income) were excluded so as to limit the scope of the review.

**Table 1:** Search Categories and Terms

**Category**	**Preliminary Search Terms**
Problem identification and agenda-setting	Advocacy, agenda, attention, collective action, commitment, framing, govern*, ideolog*, institution*, issue salience, multiple-stream*, polic*, politic*, priority, problemiti*ation
SDH and health equity	Aboriginal*, first nation*, health disparit*, health equity, health inequalit*, health inequit*, indigen*, maori, social determinants of health, social equity, social inequit*, social justice

Abbreviation: SDH, social determinants of health.

These terms were used in various combinations in different search fields so as to obtain as many relevant results as possible. Search strings were revised through preliminary searches of the included databases and assessed for specificity and sensitivity (described in the search diary). To achieve a feasible number of included studies we excluded books and grey literature. References for all studies were entered into an EndNote library and duplicates removed. To ensure comprehensiveness, three months following the original search and as analysis was about to begin, a further search was conducted to capture any additional published studies.

Quality appraisal was undertaken during the full-text screening stage. Although there is no standardised method for appraising qualitative research quality,^17,18^ best practice guidance was adopted emphasising the appropriateness of the study design, rigour in the conduct of the research, and the credibility of findings and inferences made.^17,25^ Studies were included if they had clearly described aims, explicitly stated underlying assumptions or theory used, a clearly described and justifiable study design, an appropriate methodology and data sources, a clear statement of findings, and justifiable conclusions (see inclusion criterion four). For practicality, it was decided to include only English language articles. Inclusion and exclusion criteria are listed below:

Studies were included if;

1. Published after 2000 in a peer-reviewed journal.
2. Had an abstract in English.
3. Identified factors that enabled or constrained the inclusion of health equity in government policy agendas.
4. Undertook an empirical analysis with clearly described aims, explicit underlying assumptions or theory, a clearly described study design and methodology including data sources, clear statements of findings, and justifiable conclusions.

Studies were excluded if;

1. Published prior to 2000.
2. Abstract was not in English.
3. Non-empirical (eg, editorials, commentaries, theoretical frameworks).

A flow diagram of the search results is given in Figure. PB screened all records by title, abstract and full text. To check for inter-assessor reliability AK screened a 25% (21) sample of the full-text articles, with two articles in disagreement resolved by discussion. A further reliability check was conducted whereby the 36 excluded articles were screened by all team members. This resulted in seven disagreements, resolved by discussion. An additional search three months following the original search identified one additional article. Of the 86 full text articles, 38 were excluded, resulting in 48 articles included in the final synthesis.

**Figure:**
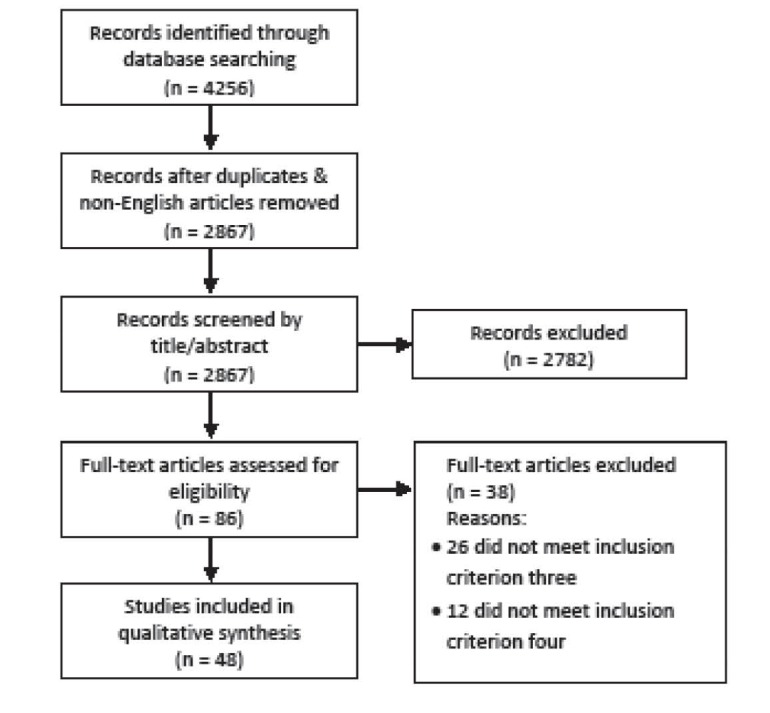
Flow Diagram of Search Results.

### Analysis

Basic data from included studies were extracted and tabulated using Microsoft Excel including study characteristics (authors, title, aims and objectives, key findings, theories used, study design, methods) and context (geographical scope, jurisdiction). To identify factors generating and impeding the inclusion of health equity in policy agendas, and to assist with data interpretation and synthesis, all articles were coded in Atlas.ti (Scientific Software Development GmbH) using a coding schema developed from the integrated theoretical framework. Free coding was also used to capture any additional factors not initially specified. The coding schema was refined using an abductive approach involving constant comparative analysis^26^ whereby the coded concepts were confirmed, modified, integrated and/or added to through several iterations of analysis. Consistent with a thematic synthesis approach, the coded data were organized into a final set of themes under the problem, policy and political streams of the theoretical framework.^27^ The coded textual data for each theme was read *in-situ* by the lead author and then synthesised. These were organized under the problem, policy and political streams described in Table 2.

**Table 2:** Integrated Framework Used to Guide the Analysis

**Problem Stream**	**Policy Stream**	**Political Stream**
Indicators Focusing events Framing the problem	Policy community cohesion Institutions Policy entrepreneurs Viable policy alternatives Framing solutions	Policy advocates Political transitions Ideology Interest group mobilization Public opinion

## Results

### Description of included studies

A total of 48 studies were included in the final review (basic data extracted from these studies are given in Text S2). The majority (76%) analysed national level jurisdictions, followed by state/provincial (12.2%), and local/municipal (10.2%). Just one study involved more than one jurisdictional level (national, state/provincial). Studies spanned 16 countries, of which 12 were high-income (Australia, the United Kingdom, Canada, England, the United States, Norway, Denmark, New Zealand, Sweden, Chile, the Netherlands, and Scotland), and three were upper-middle income (Brazil, China, and South Africa). The most studied countries were all high-income: Australia (23.3%), the United Kingdom (20%), Canada (8.3%), England (8.3%), the United States (8.3%), and Norway (6.7%).

Studies covered a diversity of policy stages and issues. The most common were policy framing and problematisation (28.6%), agenda-setting (24.5%), evidence-based policy-making (20.4%), and media framing/problemitisation (10.2%). A diversity of theoretical frameworks, theories and models were used across studies. The most common were the Kingdon’s multiple streams framework (22.6%), knowledge/evidence transfer models (11.3%), critical discourse theory (9.4%), framing/problemitisation theories (9.4%), issue salience/mediatisation theories (9.4%), and policy network theory (9.4%). Reflecting a degree of theoretical pluralism, 16 studies used a theoretical framework that integrated two or more theories.

### Factors Generating and Impeding Priority for SDH/HE in Government Agendas

#### Problem Stream

##### Framing the Problem

Issue framing influenced how the SDHI travelled into government agendas.^28-31^ Policy rhetoric typically used descriptive terms like ‘inequalities’ and ‘disparities,’ rather than the more normative (and thus potentially controversial) term ‘inequities.’ There was variation in terminology used although broader categories were identifiable, including the ‘disadvantage’ of target groups (eg, health of the poor, poor areas, poverty and disadvantage), ‘differentiation’ between population groups (eg, disparities, inequalities, dichotomies or health gaps), and ‘gradients.’^30-34^

As Gamble points out, the choice of terminology in advocacy and policy is a “matter of political strategy as well as meaning” (p. 96).^30^ The use of terminology in policy varied across the countries studied. For example, Vallgarda found that countries with residual welfare state models (Denmark and England) used ‘disadvantage’ terminology, whereas in universal welfare states it was more ‘gradients,’ as well as disadvantage (Sweden) and social exclusion (Norway). In the former there was greater emphasis on health inequalities as caused by behaviours of the disadvantaged, whereas in the latter it was more material-structural factors (eg, income, working conditions and social structures).^33^

Some studies found a shift in issue-framing as associated with changes in priority. For example, Dahl describes the emergence of priority for SDHI in Norway alongside the election of a new government and a corresponding shift in the problem definition from “individualization with a focus on health behaviours” to a “structural understanding that emphasizes the problem of the gradient and the SDH” (p. 509).^32^ In others, framing of target groups was viewed as problematic. For example, in Australia, Aboriginal and Torres Strait Islander peoples were often framed as irresponsible and incompetent at managing their health, with an underlying frame of ‘otherness.’^28^ Imagery and stories about ‘remote communities’ (ie, as distant from mainstream urban Australians) and a disproportionate use of an ‘Indigenous health crisis’ frame were used to support a conservative Government’s radical Indigenous affairs agenda.^35^

##### Indicators and Visibility

Some studies reported the SDHI becoming more publically visible, partly because of emerging data and evidence on the issue. For example, in Norway external technical publications (eg, the Black Report in the United Kingdom and WHO reports) were seen as driving a domestic research agenda, and subsequently new Norwegian data and evidence sparked public attention.^32^ Data on disparities in the health status of Aboriginal and Torres Strait Islander people was found to be an important driver of increased funding and priority for Indigenous health research.^36^ Comparative data demonstrating differences in health inequalities between national-,^32^ and among local-level,^38^ jurisdictions was found to be compelling to decision-makers in some studies.

However, Vallgarda points out that data on health inequalities were available in the United Kingdom, Denmark, and Sweden long before the issue came to receive political attention, and suggests that increasing knowledge is not crucial.^33^ Smith describes a low-level ‘appetite’ for SDHI policies among the general public, media and politicians.^39^ Gauld et al describe a low-level of awareness about population health in New Zealand government agencies outside of the health sector.^40^ In the United Kingdom, issues such as healthcare reform and waiting lists were seen to dominate the agenda more than SDHI, which ‘barely registered’ as an issue.^37,41^

### Policy Stream

#### Policy Communities

In the few cases that explicitly described the role of SDHI policy communities, most were conceived as complex actor networks with an emphasis on high-level government officials and mid-level bureaucrats.^30,37,42-44^ In some studies SDHI policy communities were non-existent or in a state of emergence. Exworthy, for example, found that civil servants and ministers in the United Kingdom had, as of 2001/2002, yet to form a policy community with “networks of information and experience” (p. 917).^37^ Others describe a ‘biomedical’ or ‘healthcare’ policy community dominated by the medical profession rather than one concerned with the SDHI.^34,43^

Few studies explicated policy communities comprising more diverse actor networks, with most tending to focus on particular actor types (eg, government officials, experts, civil society groups). One exception was Browne et al who used network analysis to describe an Aboriginal and Torres Strait Islander health policy community in Australia.^45^ This revealed a complex network with a core of Aboriginal controlled organizations and more peripheral mainstream organizations comprising medical, primary and allied health, and eye care ‘cliques’. So-called boundary spanners were actors who bridged these cliques to generate more cohesive network framing (and thus potentially greater influence).^45^

#### Civil Society and Interest Group Mobilization

Civil society mobilization was described as enabling attention and priority for SDHI in several studies.^45-49^ Advocacy was defined in one study as “the use of tools and activities that can draw attention to an issue, gain support for it, build consensus about it, and provide arguments that will sway decision-makers and public opinion to back it” (p. 72, from Rice [1999]).^48^ Strong organizational capacities (including leadership, fundraising, financial management, communication, and community engagement) were seen as important for successful social justice advocacy in South Africa,^46,47^ and Australia.^48^ However, some studies found that civil society advocacy on the SDHI can bring into play a diversity of organizations and interests and thereby the potential for fragmented advocacy efforts and weakened influence.^45,46,50^

Building a ‘base of support’ or “grassroots, leadership, and institutional support [including] the… support among the general public, interest groups, and opinion leaders,” and participation by affected groups, was found by Klugman to be a core facet of social justice advocacy (p. 152).^46^ Nathan found that ‘flexibility and opportunism’ in the use of tactics was important for influencing government (eg, working directly with policy-makers through partnerships, playing a watchdog role through the media, traditional lobbying techniques, building community support, and forming alliances and coalitions).^48^

Few studies referred to the role of other interest groups. One exception was Baum et al who found that former Australian health ministers perceived the medical profession as powerful in capturing government attention and shaping the health agenda and as ‘crowding out’ SDHI advocates in the political stream.^43^ Orton describes a similar situation in health policy reforms in the United Kingdom, where medical practitioners dominate senior positions and hold power within the system.^34^

#### Leadership

Leadership was considered critical to advancing the SDHI agenda.^30,36,37,51-53^ Gamble describes a number of leadership types, including civil rights activists and high-level government officials, as playing crucial roles in ‘catapulting’ racial and ethnic inequalities onto the political agenda in the United States.^30^ Klugman found that the election of civil society leaders into political power and government enabled the ascendance of public health and human rights issues onto the policy agenda in South Africa.^46^ Problematically, McCallum describes one individual prominent in the mainstream Australian media as the ‘singular influence’ and ‘modern face of Indigenous politics,’ who did not represent the majority views or interests of Aboriginal and Torres Strait Islander peoples (p. 145).^54^

Another study describes an influential policy entrepreneur playing a crucial role in launching homelessness onto the Canadian policy agenda.^51^ Important characteristics of this policy entrepreneur were delineated as social acuity (intuiting opportunities, and embeddedness and credibility within policy networks), effective framing (defining the problem in a way that transcended jurisdictional and partisan boundaries), working in teams (utilising repositories of skills and information within the network), and leading by example (building on past experiences and successes to build confidence and trust). Similarly SDHI policy entrepreneurs in the Dutch City of The Hague were described as “credible communicators” because of their “expertise, trustworthiness, and good will” (p. 214).^52^

Others emphasised the importance of ‘champions’ and policy entrepreneurs in enabling the successful transfer of evidence into policy.^51,53^ Pittman et al, for example, described how in the United States, China, the United Kingdom, and Chile ‘health equity champions’ within government “functioned as links between advocacy, research and policy” and helped to elevate the visibility of health equity research (p. 45).^49^

#### Institutions

Some studies recognised an important role of bureaucratic institutions in sustaining the issue on the policy agenda, and in ‘softening up’ political decision-makers (ie, making policy alternatives appear more feasible). In Norway the establishment of institutions with a public health mandate (eg, the Directorate of Health and Social Affairs), new divisions within existing departments (eg, a public health unit within a Department), technical bodies (eg, interdisciplinary expert groups), and mechanisms for community input (eg, committees and commissions), were seen as important in generating demand for action, building competencies, supporting nascent policy communities, and for formulating evidence and policy alternatives.^32,55^ In Australia, the abolishment of the Aboriginal and Torres Strait Islander Commission was described as highly detrimental to advancing the Australian Indigenous health agenda.^28,54^

However, the role of such institutions was largely constrained to the health sector. Several studies reported government institutions as important structural barriers, particularly with regards to alignment of policy agendas across sectors.^37,50,56^ In this regard, the vertical or siloed nature of government was viewed as creating a ‘formidable constraint,’^50^ and joined-up or whole-of-government approaches very difficult in practice.^42,56^ Two studies find that a health in all policies approach in South Australia avoided “health imperialism” through being sensitive to the “legitimate agendas of other sectors,” (p. 19)^57^ and alignment of this approach with broader governmental structures increased the “likelihood of influence” in the policy process (p. 1).^58^

#### Institutional Norms and Path Dependency

Several studies emphasised the influence of institutional norms (ie, the dominant belief systems and practices of policy-making institutions) as an impediment. Government agencies were in some cases described as ‘filters,’ shaping what forms of knowledge were considered legitimate in policy-making processes.^29,56^ Came, for example, describes racism as a barrier to tackling Maori HI because of it permeation throughout ‘sites’ of public health policy-making institutions in New Zealand. A degree of ‘mono-culturalism’ was found to delegitimise Indigenous knowledge, reinforced through institutional hiring practices that excluded Maori people and others with different views.^29^

A number of studies report on the rise of an ‘evidence-based policy-making’ paradigm in several countries.^28,41,44,53,59,60^ Although this was described as an attempt to ‘de-politicise’ the policy-making process, the selective uptake of public health evidence was found “to fit the political priorities of governments in power” (p. 752).^60^ Smith describes how, despite a commitment to evidence-based policy-making, government institutions in the United Kingdom acted as ‘ideational filters’ shaping the way in which evidence moved from the problemitisation and agenda-setting phases into policy. Although lifestyle-behavioural ideas had successfully made their way into policy, material-structural ideas only partially had (in rhetoric only), and other ideas were fractured (eg, psycho-social theory was adopted but not income inequality).^53,56^ Furthermore, a notable lack of ‘vertical connectivity’ within policy-making institutions and the ‘out-sourcing’ of policy advice was described as an impediment for knowledge transfer between civil servants and political decision-makers.^56^

Path dependency is often referred to as a challenge whereby policy-making institutions traditionally orientated towards hospitals and acute healthcare services, are structurally resistant to more comprehensive SDH approaches (and thus ‘path dependent’). This is consistent with a ‘lifestyle drift’ hypothesis, whereby initial commitments to tackling the ‘upstream’ material-structural determinants of health during the problemitisation and agenda-setting phases of the policy process, are reconfigured towards a ‘downstream’ approach targeting individuals and clinical services in later phases. For example, although in 2006-2007 the UK Labour government was open to “material-structural explanations” and was making “rhetorically powerful commitments,” policy was reorientated towards “culture-lifestyle behavioural approaches and medical interventions” aligned with the government’s focus on efﬁciency and patient choice.^53,59,61^

Relatedly, an institutionalised biomedical paradigm was described as highly problematic. Baum et al found that the dominance of the medical profession in policy reinforced a focus on the ‘immediacy of illness’ and acute healthcare services as solutions.^43^ Similarly, Orton found that a ‘medicalised culture within the UK health system led to SDHI approaches being ‘systematically undervalued.’^34^ Smith finds that the decision to locate responsibility for health inequalities within health departments throughout the United Kingdom reflects the “institutionalisation of a [self-perpetuating] medical model of health.”^39,56^ Furthermore, a growing emphasis of policy-making institutions on measurable ‘targets and outcomes’ may be problematic. Orton, for example, found that the introduction of a “target and outcome culture” introduced by the New Labour government in the United Kingdom “distorts priorities and further contributes to the marginalisation of the inequalities agenda” (p.8).^34^

#### Policy Alternatives and Framing Solutions

Several studies report that although there was overwhelming evidence that health inequalities exist, limited evidence on how to intervene was problematic for generating priority for SDHI.^30,56^ Exworthy finds that in the United Kingdom evidence on the ‘technical feasibility’ of policies remains limited, recommendations have been poorly communicated and not always backed by evidence.^37^ An Australian study described how solutions needed to be “broken down” and “communicated in ways that fit discretely with government departments” (p. 140).^42^

Various policy alternatives were seen as more feasible when they aligned with the ideology of government. For example, several studies found that more liberal-conservative governments were more amenable to targeted interventions and social-democratic governments to universal ones.^31,33,55,62^ Dahl and Lie report that in Norway a Christian/conservative government prioritised health inequalities when a focus on addressing poverty and social disadvantage through targeted behavioural interventions (eg, at low-income or poorly educated groups) rather than material-structural ones, was seen as fitting within that government’s ‘liberal social’ ideology.^32^ Framing solutions in ways that appeal to other sectoral interests was also important. Lawless et al, for example, suggest that early engagement in the policy process and defining health broadly as important for engaging actors from non-health sectors, described as a “shared ownership of process and product” (p. 19).^57^

In several studies, the perceived complexity of proposed policy solutions was important in shifting a government’s focus away from the SDHI.^34,35,42,43,46,51,59^ For example, Baum et al found that redistributive policies targeting the health gradient were seen as highly complex and contested in comparison to acute care services. Presenting the wider polity (and public) with “long term, complex and contested policy options” was described as politically difficult (p. 145).^43^ McCallum describes how the Howard Government in Australia came to perceive Indigenous health as an ‘intractable’ and ‘wicked’ policy problem, a contributing factor to a paternalistic policy response involving the ‘mainstreaming’ of Indigenous health and social services within portfolio departments (eg, housing, employment), under the guise of ‘practical reconciliation’ (p. 332).^35^

### Political Stream

#### Ideology

Ideology was frequently cited as an impediment to prioritising the SDHI. Adapting Smith’s (following Beland’s) definition, ideology is as an “overarching paradigm…of principles and causal beliefs providing policy-makers with organizing frameworks for understanding the world [and a] relatively coherent set of assumptions about the functioning of economic, political and social institutions” (p. 562).^39^ Neoliberalism is cited in several studies as an ideology that is highly antithetical to a SDHI agenda.^32,43,44,50,53,61^ It was viewed as methodologically individualist, emphasising efficiency driven by choice rather than equity as the core value underpinning policy.^47,53,59^ It was also perceived as a barrier to the transfer of evidence and ideas about the SDHI into policy.^53^

An individualistic, lifestyle/behavioural and/or biomedical conceptualisation of health was described as highly congruent with neoliberalism.^43,53,54,61^ McCallum, for example, describes how in Australia there was a radical shift in Indigenous policy under the Howard Government away from a “self-determinist philosophy of community control” towards a neoliberal agenda emphasising an expanded role for individual responsibility, and a highly paternalistic and militarised policy response that forced behaviour change on Aboriginal and Torres Strait Islander people (the Northern Territory Intervention) (p. 142).^54^

A neoliberal ideology was also seen as permeating public discourse and the media. Using a survey of Canadian citizens, Collins found a strong belief in assigning responsibility for health to individuals, and describes “strong ideological resistance” to income redistribution policies (p. 168).^50^ Davidson describes how the left-wing media in the United Kingdom provided the most support for government action on the SDHI, while the right-wing media tended to emphasise individual behaviours and addressed readers as ‘potential victims’ rather than the beneficiaries of policy change.^63^ In Australia, McCallum describes a similar influence of the right-wing media in promoting a ‘neo-conservative individualist’ approach to Aboriginal and Torres Strait Islander health.^35,54^

#### Changes in Political Administration

The election of new political administrations was often described as a window of opportunity for generating priority for SDHI.^28,30,43,44,47,53-56,59-61,63^ In some cases, this priority was embedded within a broader social equity agenda. For example, social equity (including health) achieved prominence on the South African policy agenda in 1994 with the post-Apartheid transition to a democratic government. Health equity achieved particular prominence because the health sector was viewed by the Government as a “vehicle for achieving rapid equity gains,” and other social policies (eg, housing, water and sanitation) were partly motivated because of their pro-equity effects (p. 1637).^47^

Several studies found that the SDHI was more likely to reach the government agenda when social-democratic governments rather than conservative (right-wing) ones were in power.^32,44,61,62^ In the United Kingdom, for example, priority for action on the SDHI is described as undergoing a series of pendulum swings as shown by government responses to the issue; a progressive 1980 Black Report on health inequalities commissioned by a social democratic Labour Government was suppressed by a Conservative Government shortly after, followed by the 1998 Acheson report and 2008 Marmot review under respective Labour governments, with the latter ignored by a newly elected Conservative Government in 2010.^61^ However, despite reaching the agenda of the Labour government in 2006-2007, a neoliberal ideology was described as the ultimate political ‘block’ or ‘veto’ on the adoption of a comprehensive SDHI approach in policy.^53^

In contrast, in several of the Scandinavian social democracies the SDHI agenda was described as being more resilient to different forms of (and changes in) political administration.^31-33^ This resilience was seen to reflect the strong non-partisan commitment to reducing inequalities and redistribution as foundational tenets of the social democratic welfare state model.^31-33,55,62^

## Discussion

This review has identified factors enabling and constraining the inclusion of health equity in government policy agendas based on extant empirical evidence, and we find, by and large, strong support for the theoretical formulations of Kingdon and Shiffman and Smith. The empirical findings reviewed support the view that the processes by which SDHI issues enter government agendas are shaped by a complex set of interrelated and context-dependent factors across problem, policy and political streams and no one single factor predominates. A diversity of theoretical frameworks, theories and models have been used across the studies, providing multiple lenses through which to understand this complex topic.^21^ Although the majority of included studies were only indirectly comparable (ie, due to differences in theoretical and methodological approaches), there was little consistency across studies with regard to which combination of factors mattered most. Thus it is better to think about the identified factors as increasing (or decreasing) the ‘probability’ of SDHI reaching an agenda, rather than in terms of ‘necessity’ or ‘sufficiency.’^11^ In the following section we present and interpret several key findings of the review.

The most consistent and significant findings are from studies on the role of framing, ideology and institutional norms. Although SDHI issues have ascended onto public policy agendas across a diversity of country-contexts and under governments of different ideological persuasions, how they are problemitised as they enter agendas (and into policy) has varied significantly. SDHI problems have been framed in terms of *disadvantage* (eg, of the poor, or of indigenous groups), *differentiation* (eg, disparities, inequalities and health gaps between groups), and *gradients* (eg, distributions across populations). These are not just evidence-based descriptors; they are also inherently political terms, because they infer different causes and solutions, and attributions of responsibility that are more or less congruent with ideologies inherent to different welfare state models (eg, residual vs. universalist) and/or governments (eg, social-democratic vs. liberal-conservative).

Several deeply embedded and inter-connected belief systems within public, media and policy-making institutions were identified as having powerfully impeded the inclusion of SDHI in policy agendas: a *neoliberal ideology* emphasising individualism, economic rationalism and efficiency over equity; a *biomedical paradigm* emphasising a medical rather than social orientation to health; and, in some cases, pervasive *racism*. Although there is a significant evidence-base describing SDHI problems, the solutions to these problems are often perceived as having a limited supporting evidence base, as technically and politically infeasible, and as misaligned with existing policy preferences and institutional arrangements. And although the SDHI may be included on government agendas, evidence in support of different causal theories (eg, lifestyle-behavioural, psychosocial and material-structural interpretations) may be selectively ‘filtered’ to align with the ideological preferences of government as issues travel from the agenda-setting phase and on into policy.

To overcome these challenges, a small number of studies suggest greater emphasis on moral and dialogic as well as technocratic engagement by advocates with policy-makers, and framing SDHI to align better with existing institutions and policy arrangements, and with the ideologies of those in power.^30,42,43^ However, the literature offers little insight into the efficacy of these strategies, nor practical guidance on how to implement them. A strong emphasis of the literature on the role of ideas in policy has generated important insights, but it has (with some exceptions) deemphasised the obstructive role of power emanating from particular interest groups. For example, biomedicine as an impediment to advancing the SDHI agenda is described largely as an ideological constraint (eg, a medicalised culture) rather than one relating to the power of the medical profession. The small number of studies emphasising the important enabling role of civil society groups suggest that advocacy to generate priority is likely to require flexibility and dynamism, the development of strong organizational capacities and draw upon a diversity of strategies and tactics.

The integrated theoretical framework used to guide the review identified many but not all of the factors evident in the literature. For example, neither Kingdon’s multiple stream’s theory nor Shiffman’s priority-setting framework give due consideration to the role of the media in framing policy issues. Nor do they adequately account for the complex institutional arrangements (ie, multi-sector and multi-level). The corollary is that several factors identified in these frameworks were not evidenced in the literature reviewed. For example, policy communities were often poorly defined entities, typically consisting of government actors. This is a potential weakness of the field given that contemporary conceptualisations of governance (eg, network governance) emphasise the roles of non-government as well as government actors. Furthermore, focusing events that generate or constrain attention to and/or priority for the SDH/HE were almost exclusively described as changes in government (ie, with priority more likely under social-democratic and egalitarian governments than liberal-conservative ones). Although potentially of significant importance, the role of other social and/or economic phenomena (eg, changing economic conditions, rising inequality) have appeared infrequently in the literature.

This review has several limitations. We have not elaborated on how the reported factors interact (particularly factor-mechanism-context interactions), even though such interactions are likely to be important they are beyond the scope of this review and they were rarely explicitly studied empirically. The large majority of studies were focused on a small number of high-income countries, and thus we did not delineate the findings based upon country income-status or other characteristics. Furthermore, our focus on the early stages of the policy process does not account for interactions (eg, feedback loops) with later stages. For example, the failure to implement effective programmes can weaken government commitment to an issue in the long-term. Additionally, this review has focused on understanding the ‘government agenda’ and not the ‘systemic agenda’ – the list of subjects that are often prominent in the media, and in the broader ‘polity’ as topics of societal discussion and debate.^64^ Thus, this review may have deemphasised the role of public opinion, the media, and other societal-normative influences. By giving emphasis to Indigenous search terms we may have over-emphasised Australian studies, partly because this is a key, current issue for Australian health policy. Never-the-less, there are many nations in the developed and developing world where Indigenous health issues are of concern, even if neglected in the research.

## Conclusion

What factors have enabled and constrained the inclusion of the SDHI in government policy agendas? Guided by political science theories of the policy process this review revealed that a complex set of interrelated factors are influential, including *inter alia* the ways in which the SDH and HI are framed in political discourse, the role of institutions, norms and underlying ideologies in shaping which forms of evidence and ideas are adopted and which are not, the mobilization of civil society, strong leadership, and the election of social-democratic governments. Understanding the factors that influence government agenda-setting may help advocates develop better strategies for influencing decision-makers and generating priority for health equity in the future.

## Acknowledgements

This work was supported by the NHMRC Centre of Research Excellence on the Social Determinants of Health Equity: Policy research on the social determinants of health equity (APP1078046). The NHMRC had no role in the conduct of this research.

## Ethical issues

Not applicable.

## Competing interests

Authors declare that they have no competing interests.

## Authors’ contributions

All authors conceived the idea for the review through group discussion. PB undertook the systematic search, extracted the data including coding of the included studies, analysed the data, and wrote the first draft of the manuscript. All authors provided input into ongoing iterations of the manuscript and approved the final version.

## Authors’ affiliations

^1^Institute for Physical Activity and Nutrition, School of Exercise and Nutrition Sciences, Deakin University, Geelong, VIC, Australia. ^2^School of Regulation and Global Governance (RegNet), College of Asia and the Pacific, Australian National University, Canberra, Australia. ^3^Institute of Policy Studies, University Brunei Darussalam, Gadong, Brunei Darussalam. ^4^Southgate Institute of Health, Society and Equity, Flinders University, Adelaide, SA, Australia. ^5^National Centre for Epidemiology and Population Health, College of Medicine, Biology & Environment, Australian National University, Canberra, Australia.

## References

[1] Commission on Social Determinants of Health. Closing the gap in a generation: health equity through action on the social determinants of health. Geneva: World Health Organization; 2008. 10.1016/S0140-6736(08)61690-6 18994664

[2] Lalonde M. A new perspective on the health of Canadians: a working document. Ottawa: Ministry of National Health and Welfare; 1974.

[3] Mackenbach JP (2012). The persistence of health inequalities in modern welfare states: the explanation of a paradox. Soc Sci Med.

[4] Exworthy M (2008). Policy to tackle the social determinants of health: using conceptual models to understand the policy process. Health Policy Plan.

[5] Embrett MG, Randall GE (2014). Social determinants of health and health equity policy research: exploring the use, misuse, and nonuse of policy analysis theory. Soc Sci Med.

[6] Farrer L, Marinetti C, Cavaco YK, Costongs C (2015). Advocacy for health equity: a synthesis review. Milbank Q.

[7] Buse K, Mays N, Walt G. Making health policy. Open University Press; 2012.

[8] Parkhurst JO. The Politics Of Evidence: From Evidence-Based Policy to the Good Governance Of Evidence. Abingdon; New York: Routledge; 2017.

[9] Cairney P. The Politics of Evidence-Based Policy Making. London: Palgrave Pivot; 2016.

[10] Kingdon JW. Agendas, Alternatives, and Public Policies. London: Harper Collins; 1995.

[11] Shiffman J, Smith S (2007). Generation of political priority for global health initiatives: a framework and case study of maternal mortality. Lancet.

[12] World Health Organization. Closing the Gap: Policy Into Practice on Social Determinants of Health. Rio de Janeiro: WHO; 2011.

[13] Lasswell HD. Politics: Who Gets What, When, How. New York: P Smith; 1950:1.

[14] Graham H (2004). Graham HSocial determinants and their unequal distribution: clarifying policy understandings. Milbank Q.

[15] Parsons W. Public policy. Cheltenham, Northampton: Edward Elgar; 1995.

[16] Shiffman J (2009). A social explanation for the rise and fall of global health issues. Bull World Health Organ.

[17] Petticrew M, Roberts H. Systematic reviews in the social sciences: A practical guide. John Wiley Sons; 2008.

[18] Popay J, Roberts H, Sowden A, et al. Guidance on the conduct of narrative synthesis in systematic reviews: A product from the ESRC methods programme. Lancaster University; 2006.

[19] Centre for Research Excellence in the Social Determinants of Health Equity. http://www.flinders.edu.au/medicine/research/centres/centre-for-research-excellence-in-the-social-determinants-of-health-equity/. Accessed December 6, 2012.

[20] Anderson I, Robson B, Connolly M (2016). Indigenous and tribal peoples’ health (The Lancet-Lowitja Institute Global Collaboration): a population study. Lancet.

[21] Sabatier P. The need for better theories. In: Sabatier P, Weible C, eds. Theories of the Policy Process. Boulder, CO: Westview Press; 2014.

[22] World Health Organization. Closing the gap in a generation: health equity through action on the social determinants of health. Geneva; WHO; 2008. 10.1016/S0140-6736(08)61690-6 18994664

[23] Krieger N (2001). A glossary for social epidemiology. J Epidemiol Community Health.

[24] Kawachi I, Subramanian SV, Almeida-Filho N (2002). A glossary for health inequalities. J Epidemiol Community Health.

[25] Spencer L, Ritchie J, Lewis J, Dillon L. Quality in qualitative evaluation: a framework for assessing research evidence. Government Chief Social Researcher’s Office; 2003.

[26] Corbin J, Strauss A. Basics of Qualitative Research: Techniques and Procedures for Developing Grounded Theory. 4th ed. San Jose State University, USA: SAGE Publishing; 2008.

[27] Thomas J, Harden A (2008). Methods for the thematic synthesis of qualitative research in systematic reviews. BMC Med Res Methodol.

[28] Aldrich R, Zwi AB, Short S (2007). Advance Australia fair: social democratic and conservative politicians’ discourses concerning Aboriginal and Torres Strait Islander Peoples and their health 1972-2001. Soc Sci Med.

[29] Came H (2014). Sites of institutional racism in public health policy making in New Zealand. Soc Sci Med.

[30] Gamble VN, Stone D (2006). US policy on health inequities: the interplay of politics and research. J Health Polit Policy Law.

[31] Vallgårda S (2007). Health inequalities: Political problematizations in Denmark and Sweden. Crit Public Health.

[32] Dahl E, Lie M (2009). Policies to tackle health inequalities in Norway: from laggard to pioneer?. Int J Health Serv.

[33] Vallgarda S (2008). Social inequality in health: dichotomy or gradient? A comparative study of problematizations in national public health programmes. Health Policy.

[34] Orton LC, Lloyd-Williams F, Taylor-Robinson DC, Moonan M, O’Flaherty M, Capewell S (2011). Prioritising public health: a qualitative study of decision making to reduce health inequalities. BMC Public Health.

[35] McCallum K (2013). Distant and intimate conversations: media and indigenous health policy in Australia. Crit Arts.

[36] de la Barra SL, Redman S, Eades S (2009). Health research policy: a case study of policy change in Aboriginal and Torres Strait Islander health research. Aust New Zealand Health Policy.

[37] Exworthy M, Blane D, Marmot M (2003). Tackling health inequalities in the United Kingdom: the progress and pitfalls of policy. Health Serv Res.

[38] McGill E, Egan M, Petticrew M (2015). Trading quality for relevance: non-health decision-makers’ use of evidence on the social determinants of health. BMJ Open.

[39] Smith KE (2014). The politics of ideas: The complex interplay of health inequalities research and policy. Sci Public Policy.

[40] Gauld R, Bloomfield A, Kiro C, Lavis J, Ross S (2006). Conceptions and uses of public health ideas by New Zealand government policymakers: report on a five-agency survey. Public Health.

[41] Exworthy M, Berney L, Powell M (2002). ‘How great expectations in Westminster may be dashed locally’: the local implementation of national policy on health inequalities. Policy Polit.

[42] Carey G, Crammond B (2015). Action on the social determinants of health: views from inside the policy process. Soc Sci Med.

[43] Baum FE, Laris P, Fisher M, Newman L, Macdougall C (2013). “Never mind the logic, give me the numbers”: former Australian health ministers’ perspectives on the social determinants of health. Soc Sci Med.

[44] Smith KE (2015). Understanding responses to the political context of health inequalities in research and policy: Can post-structural theories of power help?. Soc Theory Health.

[45] Browne J, de Leeuw E, Gleeson D, Adams K, Atkinson P, Hayes R (2017). A network approach to policy framing: A case study of the National Aboriginal and Torres Strait Islander Health Plan. Soc Sci Med.

[46] Klugman B (2011). Effective social justice advocacy: a theory-of-change framework for assessing progress. Reprod Health Matters.

[47] McIntyre D, Gilson L (2002). Putting equity in health back onto the social policy agenda: experience from South Africa. Soc Sci Med.

[48] Nathan S, Rotem A, Ritchie J (2002). Closing the gap: building the capacity of non-government organizations as advocates for health equity. Health Promot Int.

[49] Pittman PM (2006). Beyond the sound of one hand clapping: Experience in six countries using health equity research in policy. J Health Polit Policy Law.

[50] Collins PA, Abelson J, Eyles JD (2007). Knowledge into action? understanding ideological barriers to addressing health inequalities at the local level. Health Policy.

[51] Macnaughton E, Nelson G, Goering P (2013). Bringing politics and evidence together: policy entrepreneurship and the conception of the At Home/Chez Soi Housing First Initiative for addressing homelessness and mental illness in Canada. Soc Sci Med.

[52] Schmidt M, Joosen I, Kunst AE, Klazinga NS, Stronks K (2010). Generating political priority to tackle health disparities: a case study in the Dutch city of The Hague. Am J Public Health.

[53] Smith KE (2007). Health inequalities in Scotland and England: the contrasting journeys of ideas from research into policy. Soc Sci Med.

[54] McCallum K, Waller L (2013). The Intervention of Media Power in Indigenous Policy-Making. Media International Australia.

[55] Strand M, Fosse E (2011). Tackling health inequalities in Norway: applying linear and non-linear models in the policy-making process. Crit Public Health.

[56] Smith K (2013). Institutional filters: The translation and re-circulation of ideas about health inequalities within policy. Policy Polit.

[57] Lawless AP, Williams C, Hurley C, Wildgoose D, Sawford A, Kickbusch I (2012). Health in All Policies: evaluating the South Australian approach to intersectoral action for health. Can J Public Health.

[58] Delany T, Harris P, Williams C (2014). Health impact assessment in New South Wales & Health in All Policies in South Australia: differences, similarities and connections. BMC Public Health.

[59] Qureshi K (2013). It’s not just pills and potions? depoliticising health inequalities policy in England. Anthropol Med.

[60] Nutbeam D, Boxall AM (2008). What influences the transfer of research into health policy and practice? Observations from England and Australia. Public Health.

[61] Bambra C, Smith KE, Garthwaite K, Joyce KE, Hunter DJ (2011). A labour of Sisyphus? Public policy and health inequalities research from the Black and Acheson Reports to the Marmot Review. J Epidemiol Community Health.

[62] Fosse E (2009). Norwegian public health policy: revitalization of the social democratic welfare state?. Int J Health Serv.

[63] Davidson R, Hunt K, Kitzinger J (2003). ‘Radical blueprint for social change’? Media representations of New Labour’s policies on public health. Sociol Health Illn.

[64] Cobb RW, Elder CD. Participation in American politics: The Dynamics of Agenda-Building. Johns Hopkins University Press; 1983.

